# Machining Strategy Determination for Single- and Multi-Material Wire and Arc Additive Manufactured Thin-Walled Parts

**DOI:** 10.3390/ma16052055

**Published:** 2023-03-02

**Authors:** Ozan Can Ozaner, Damjan Klobčar, Abhay Sharma

**Affiliations:** 1 Department of Materials Engineering, Faculty of Engineering Technology, KU Leuven, Campus De Nayer, 2860 Sint-Katelijne Waver, Belgium; 2 Faculty of Mechanical Engineering, University of Ljubljana, Aškerčeva 6, 1000 Ljubljana, Slovenia

**Keywords:** bimetallic, specific cutting energy, machinability, WAAM, surface integrity

## Abstract

Wire and arc additive manufacturing (WAAM) technology has recently become attractive due to the fact of its high production capacity and flexible deposition strategy. One of the most prominent drawbacks of WAAM is surface irregularity. Therefore, WAAMed parts cannot be used as built; they require secondary machining operations. However, performing such operations is challenging due to the fact of high waviness. Selecting an appropriate cutting strategy is also challenging, because surface irregularity makes cutting forces unstable. The present research determines the most suitable machining strategy by assessing the specific cutting energy and local machined volume. Up- and down-milling are evaluated by calculating the removed volume and specific cutting energy for creep-resistant steel, stainless steel, and their combination. It is shown that the main factors that affect the machinability of WAAMed parts are the machined volume and specific cutting energy rather than the axial and radial depths of the cut due to the fact of high surface irregularity. Even though the results were unstable, a surface roughness of 0.1 µm was obtained with up-milling. Despite a two-fold difference in the hardness between the two materials in the multi-material deposition, it is found that hardness should not be used as a criterion for as-built surface processing. In addition, the results show no machinability difference between multi- and single-material components for a low machined volume and low surface irregularity.

## 1. Introduction

The production of parts via additive manufacturing is becoming widespread in various industries. These parts are used as built or are subjected to secondary processes, such as machining and rolling. There are numerous additive manufacturing methods. Wire and arc additive manufacturing (WAAM) is often preferred due to the fact of its large and fast production capacity and flexible deposition strategy [1]. However, the surface irregularity of as-built WAAMed parts is higher than those made using other additive processes and conventional manufacturing methods. Compared to other processes, WAAM shows poor results in terms of waviness, flatness [2,3], and other surface roughness criteria [4]. Therefore, secondary machining operations are required for WAAMed parts [5]. In the aviation industry, additive manufacturing has been adopted to minimize the required secondary processing [6]. Even parts manufactured using selective laser melting, which can produce much better surfaces than those obtained with WAAM, are subjected to secondary processing due to the fact of very narrow tolerances [7]. The machinability of WAAMed parts has thus received research attention.

In WAAM, flatness/waviness is mostly due to the fact of high heat input and low cooling rates [5,8]. High surface irregularity has a negative impact on machining. Generally, the initial surface state influences the final roughness [8] and can cause unstable cutting conditions, leading to unwanted conditions such as forced vibration [8,9]. High surface irregularity causes variation in the depth of the cut in the cut direction. An increase in the depth of the cut in the process direction due to the fact of surface variability can even result in tool breakage [5]. An increase in the surface irregularity increases the runout of as-built parts and complicates force analysis [10]. Runout is not the only factor that makes force examination difficult. Due to the frame used in additive manufacturing, the melt pool shape varies according to the parameters and cooling rates. Therefore, material anisotropy, which can cause force variation, can be observed. Forces are more stable in processing cast and forged parts [11].

Due to the high surface irregularity, the depth of cut must be determined for evaluating the machinability of WAAMed parts. However, in contrast to the semi-finished and finished cuts for cast and forged geometries, a constant depth of cut cannot be determined. To determine the depth of cut, WAAMed parts are usually 3D scanned, and the machining allowances are calculated by fitting the scanned models to the target model [8,9]. Some studies removed the specified machining allowances in a single pass [8] or pre-machined high-waviness surfaces and then machined the subsurfaces in multiple passes [9,12]. The cutting forces are unstable in the first pass due to the surface irregularity for the multi-pass strategy. In subsequent passes, because the surface waviness has been removed, the processing is conducted under stable conditions, as is the case for casting and forging parts [9].

Surface waviness is directly related to the WAAM parameters. Optimization studies have confirmed that WAAM parameters significantly affect the machinability; for example, a higher heat input increases the surface irregularity [8,9]. Moreover, higher heat input and lower cooling rates increase the surface waviness of parts and decrease the hardness [2,8,10]. According to the general machining methodology, high hardness causes high cutting forces [5,8]. However, no specific influence of hardness on the machinability of low-carbon steels produced with WAAM has been found [13]. Nevertheless, the stiffness of parts decreases due to the changes in cooling rates, causing unwanted conditions, such as chatter during machining [1].

Once the cutting strategy has been selected, the basic milling parameters must be set. It has been reported [1,8,9] that the axial depth of the cut should be minimized to achieve the best surface quality [1,8]. As a general rule, the surface quality obtained by milling deteriorates as the irregularity of the component surface increases. Nevertheless, relatively good surface quality can be achieved despite high surface irregularity by machining with a small axial depth of cut [8]. However, by optimizing the parameters, such as cutting speed and feed rate, rather than the axial depth of cut, parts with similar surface quality can be obtained by machining both high and low surface irregularities [9]. Furthermore, it is suggested that the cutting direction should have the minimum waviness [9]. Similar results have been reported for the cutting speed and feed rate for machining cast and forged materials [4,8,9,12]. Thus, studies have generally focused on up- and down-milling, not parameter variation [10,12]. According to a general evaluation of machining strategies, up-milling produces a worse surface quality than that obtained with down-milling; however, in some cases, up-milling produces better surface quality [12,14]. These contradictory results indicate that this issue needs further investigation in the context of WAAM.

Studies on WAAM are generally limited to single-material deposits and their surface properties. Few studies have been conducted on producing multi-material components, even though the demand for such products has increased [15,16,17,18,19]. However, studies are generally limited to examining the appropriate parameter selection [17,18] or basic structural integrity criteria [19] for multi-material deposition. There is thus a large research gap regarding the machinability of multi-material WAAMed parts. For single-material processing, the precise routing and the processing procedure are still unclear. Although some studies have been conducted on the machinability of WAAM workpieces, they focused on the variation of the depth of cut due to the fact of surface irregularity. In the present study, a guideline was developed for the machinability of WAAM workpieces, focusing on the actual machined local volume, as the evaluation of the depth of cut in the literature is insufficient. Since the literature is unclear regarding the most suitable cutting strategy, this study also investigated the determination of a suitable one. In addition, there is some uncertainty regarding the machinability of multi-material WAAMed parts. To the best of the authors’ knowledge, previous studies on the machinability of WAAMed parts focus exclusively on single-material parts. Various materials have been used in WAAM. Among them, stainless steel (SS) and creep-resistance steel (CRS) are preferred for large and complex thin-walled geometries [20]. In addition, since WAAM is used for repair in many industries, the deposition process conditions and results for SS and CRS, both individually and their combination, in WAAM should be clarified [21]. Therefore, the present study focused on the actual machined volume and not the depth of cut for evaluating the machinability of single- and multi-material components made of SS and CRS.

## 2. Materials and Methods

### 2.1. Experimental Procedure for Wire and Arc Additive Manufacturing

**Figure 1:**
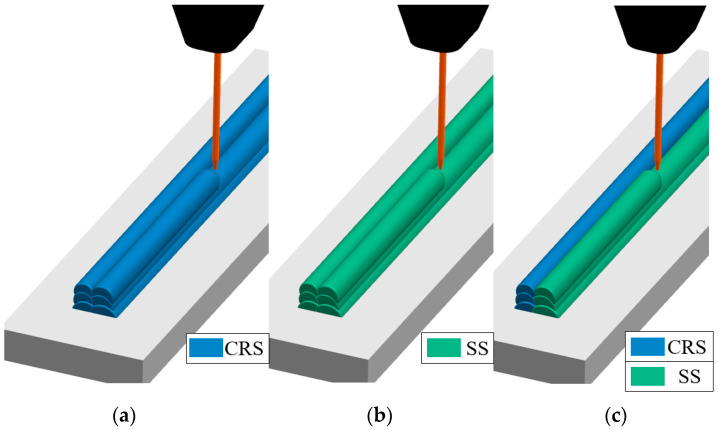
Diagrams of the deposition strategy for (**a**,**b**) single- and (**c**) multi-material.

**Figure 2:**
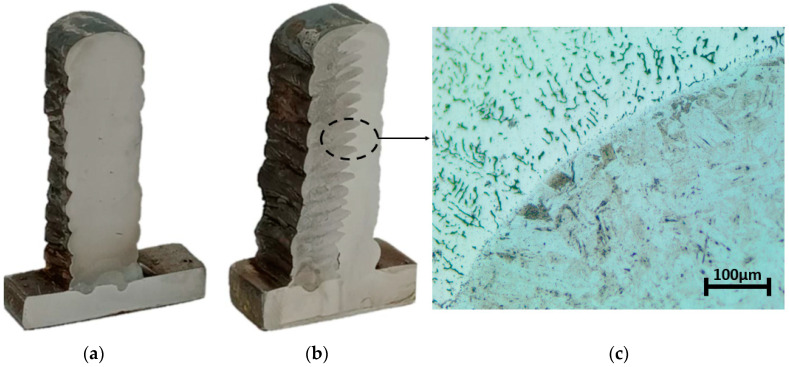
Representative sections of (**a**) single-material and (**b**) multi-material deposition; (**c**) intersection in multi-material.

Two power sources (TransPuls Synergic 4000, Fronius International GmbH, Wels, Austria) and a twin torch mounted on a six-axis robotic arm (UR10e, Universal Robots A/S) were used in this study. The filler metal was 1.2 mm diameter solid-wire electrodes made of SS and CRS. The material compositions are listed in Table 1. A 304 SS plate with the dimensions of 100 mm × 30 mm × 9 mm was used as the substrate. A mixture of Ar, 3% CO_2_, and %1 O_2_ (trade name: M14) with a constant flow rate of 24.0 l/min was used as the inert gas. Thin walls were deposited as several layers in a double pass, as shown in Figure 1. As shown, both single- and multi-material thin walls were deposited. Representative samples of single- and multi-material deposition are shown in Figure 2. The deposition follows a zig-zag course to compensate for the deviation of the start and end points of the bead shape, as conducted in similar studies [8,9]. For the deposition methods shown in Figure 1, different WAAM parameter values were used for the materials. The parameters were adjusted to avoid a lack of fusion and sufficient penetration by minimizing the heat input [15]. The heat input (*HI*) was calculated for each case using Equation (1), where *k* is the thermal efficiency factor of a specific welding process (*k* = 0.8, as per EN 1011-1), *V* is the arc voltage, *I* is the welding current, *TS* is the travel speed, *H* is the bead height, and *W* is the bead width.

(1) $$HI\left(\frac{kJ}{mm^{3}}\right)=k\times \frac{V\left(V\right)\times I\left(A\right)}{TS\left(\frac{mm}{s}\right)\times H\left(mm\right)\times W\left(mm\right)}\times 10^{3}$$

The parameters used in this investigation and the calculated values for heat input are shown in Table 2. At the end of the deposition, thin-walled geometries with approximate dimensions of 40 × 80 mm (height × length) were obtained. The interpass temperature was kept at 110 °C in all cases. After the deposition of a layer, the specified interlayer temperature was checked with a Mastech MS6520B pyrometer with a 0.1 °C resolution. The next layer was deposited after the selected interpass temperature had been reached.

**Figure 3:**
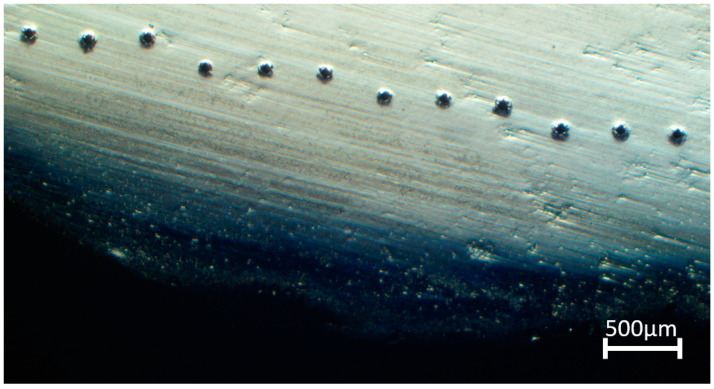
Representative image of indentations used for the hardness measurements.

After deposition, a low-force Vickers hardness test (HV1) based on EN ISO 6507-1 was performed on the parts, as shown in Figure 2. The specimens were prepared by grinding and polishing with 3 mm diamond powder. The tests were implemented on a micro-hardness measurement machine (HMV-2000, Shimadzu Corporation, Tokyo, Japan) with a 1 kgf load applied for 13 s. In this study, since the machinability of single- and multi-material deposited WAAMed parts were compared, as shown in Figure 3, the hardness measurement was conducted three times at the edges, with 300 μm between adjacent indentations. The average hardness along a bead was thus examined. The indentations were measured with a digital microscope (KH-8700, Hirox Corporation, Tokyo, Japan).

### 2.2. Experimental Procedure for Milling

**Figure 4:**
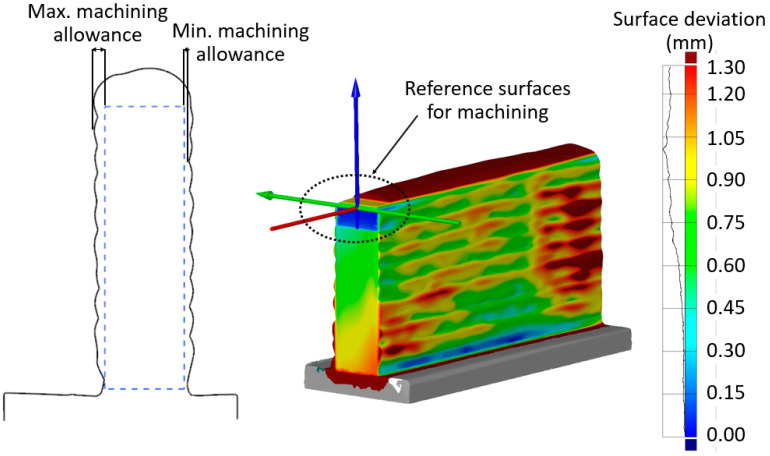
Pre-alignment used for the machining allowance calculation and a 3D model showing the maximum and minimum deviations.

High heat input during WAAM distorts the substrate [8,9,10]. Fixturing for the milling and the actual position of the deposited part are uncertain due to the fact of this distortion. Substrate subsurfaces are thus often pre-machined [8,9,10] to facilitate the fixturing for the milling and to improve the positioning accuracy. Then, the entry and exit areas of the part are removed using pre-machining until the irregularity is eliminated. The irregular geometric shape at the start and end points during deposition would have a negative effect on milling. In addition, the upper surface of the part is pre-machined and flattened, and the preparations necessary for a stable milling process are completed. These pre-machining operations prevent critical tool engagement and prolonged cuts without material removal [9]. In the present study, reference surfaces were created on the part during the pre-machining operations to define the tool paths, as shown in Figure 4. The tool paths required for machining the target areas for milling were defined using these reference surfaces. The milling parameter values, which depend on the workpiece and tool material, were selected based on the tool catalogue provided by the tool manufacturer. Table 3 shows the milling parameters used in this study.

After the necessary pre-machining, the parts were scanned with a 3D scanner (ATOS Compact Scan, GOM Metrology). The results are shown in Figure 4. The machining allowances were determined by fitting the target geometry into the scanned geometry data to the shape parallel to the substrate side surfaces. The specified machining allowances were applied as the radial depth of cut and machining in a single pass was designed. The calculated machining allowances are shown in Table 4. The machining strategies and experimental settings are shown in Table 5.

**Figure 5:**
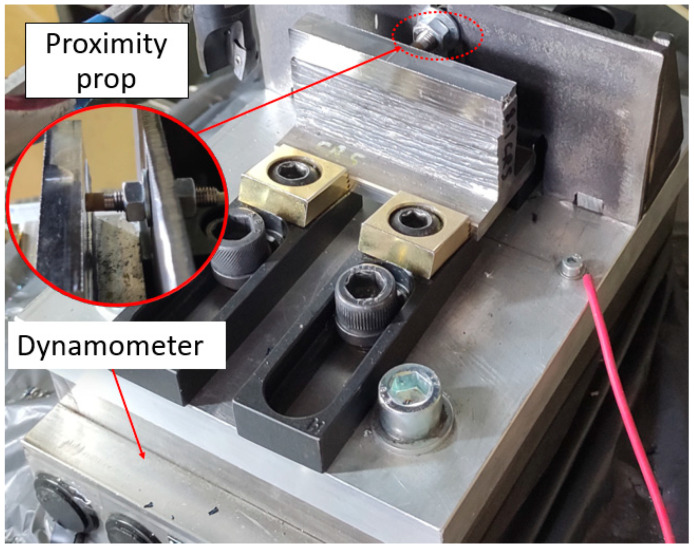
Experimental setup for the milling of WAAMed thin-walled components.

A five-axis machine center (DMU 50, DMG MORI) and a square-shoulder end mill (R390-016A16-11L, Sandvik) with exchangeable inserts (R390-11T3 08M-PM 4330 (carbide-coated-CVD TiCN + Al2O3 + TiN); diameter: 16 mm, helix angle: 15, number of teeth: 2) were used for milling. A three-axis dynamometer (Type 9257B, Kistler) was used during machining. The sampling frequency was 20,000 Hz. In addition, as shown in Figure 5, the part deflection was recorded using the proximity probe during the machining. A transducer system (7200 Proximity, Bently Nevada) was used as a probe. The displacement signals were acquired using a data acquisition system (NI 6361, National Instruments) with a 50 kHz sampling frequency. The milling was performed in dry conditions. 

### 2.3. Measurement Strategy

**Figure 6:**
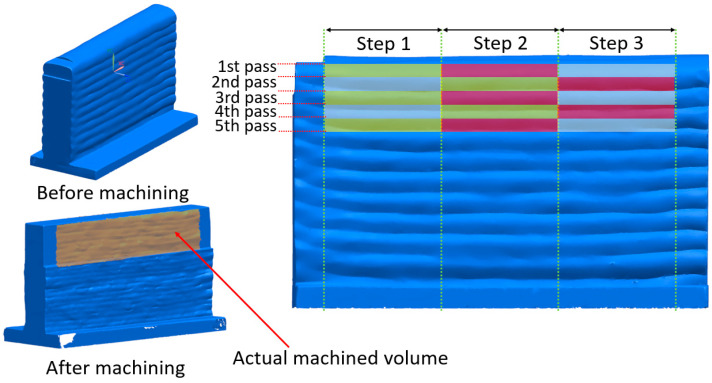
Division of a 3D scanned workpiece into equal regions for the calculation of the actual machined volume.

After the machining operations, each pass interval was measured five times with a contact-based profilometer (Formtracer CS-3200, Mitutoyo America Corporation, Aurora, Illinois, America). The average values were used in the analyses. Since the force and displacement data, explained in more detail in the subsections below, did not provide meaningful results, a new methodology was developed. The force and surface roughness results were examined by dividing the parts into three regions, as shown in Figure 6, with a minimum inspection length of 20 mm. The machined volume was calculated accordingly. However, thermal-induced warpage caused distortion. In addition, the target tool path could not be completed, and the target area could not be fully machined due to the fact of unwanted conditions, such as chatter vibration [22]. Therefore, the machined parts were scanned again. The machined volume was calculated by subtracting the post-machining geometry from the pre-machining geometry using mesh analysis software (GOM Inspect v2.0.1) and computer-aided design software (Siemens NX v8.5).

**Figure 7:**
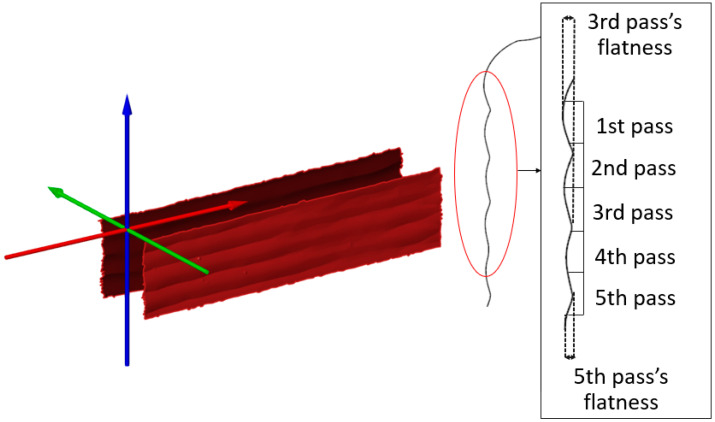
Surface irregularity calculation and demonstration of flatness at various passes.

Since the surface irregularity before machining affects the force data, the surface irregularity of the areas to be machined was calculated for each pass. The results are shown in Figure 7. As shown, different surface irregularity values were obtained in each pass. The surface irregularity was calculated using sections of 3D scanning data at 0.01 mm intervals.

## 3. Results and Discussion

In this study, single-material (SS or CRS) and multi-material (SS + CRS) thin-walled parts produced via WAAM were milled. The correlations between the force data and surface roughness were obtained. A methodology for selecting up- or down-milling, the depth of cut for the cutting strategies, and the required cutting direction for the tool path were developed. Analyses of the force data, actual machined volume, surface irregularity, and displacement data were carried out. In addition, the hardness of each sample was measured, and the relationship between hardness and machinability was examined.

### 3.1. Evaluation of Cutting Forces

Several methods exist for examining force data [3,13,19,20]. The forces Fx (axial) and Fy (radial), both of which are considered to be active, are frequently evaluated, whereas Fz, a passive force, is rarely included in evaluations [3]. Fx and Fy cause the deflection of the workpiece and the tool and are thus considered in many studies [13,23,24]. Moreover, the Fz force is considerably lower than Fx and Fy in thin-wall machining [23]. This was confirmed in the present study. Fx and Fy were thus selected for calculating the resultant forces.

**Figure 8:**
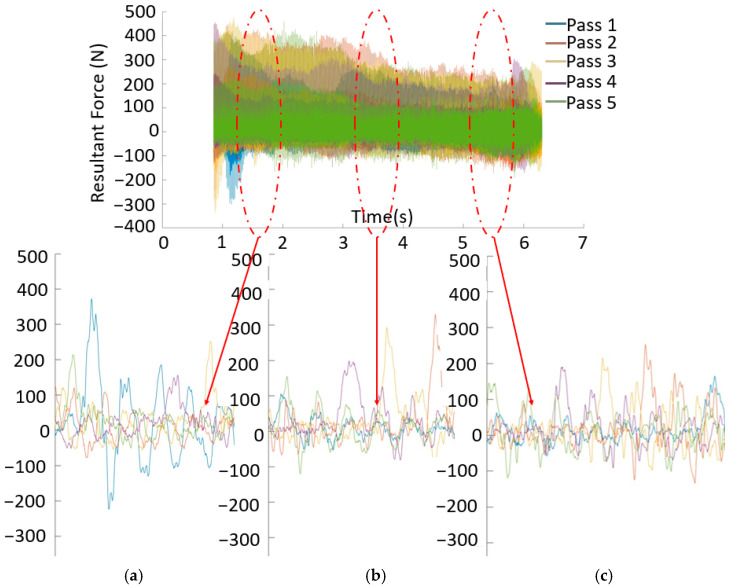
Evaluation of the resultant forces for the time intervals of (**a**) 1.12 to 1.13 s; (**b**) 3.52 to 3.53 s; (**c**) 5.45 and 5.46 s for case number 4.

The obtained forces were examined by determining the resultant force values. However, as shown in Figure 8, the force data were unstable. This situation was expected due to the surface irregularity of WAAM workpieces [9]. To obtain stable forces, the waviness of the surface must be machined as a pre-machining operation. However, the parts were machined directly to their final dimensions by adopting the buy-to-fly ratio as a target measure [6]. The variation of forces after each pass was measured. The results of the forces collected during five passes for case number 4 are shown in Figure 8. An examination of the three sections, shown in Figure 8a–c, reveals that the forces were constantly changing. For the machining of thin-walled parts, much higher or lower forces are expected in the passes near the fixed end compared to those in the first few passes. However, this trend was not observed due to the force variation caused by surface irregularities.

**Figure 9:**
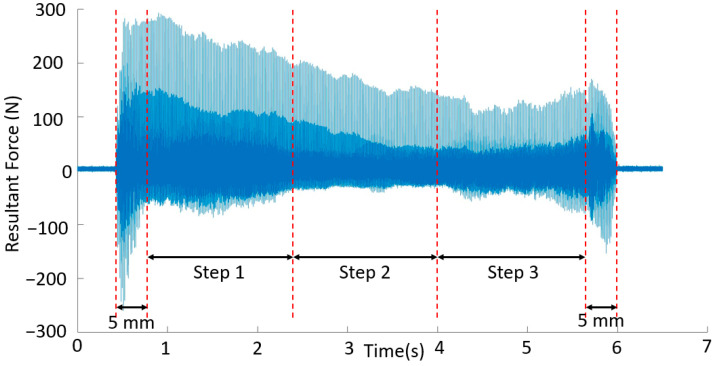
Division of the resultant force into regions for interpretation (case number 4, 3rd pass).

The machining allowances were not constant, as shown in Figure 4. Therefore, the machined volume varies due to the high surface irregularity, even in a single machining pass. It is known that a high volume increases the resultant force [13]. The force data for each pass were thus divided into three regions, as shown in Figure 9. The first and last 5 mm, where the tool enters and exits the part, respectively, were excluded from the investigation so that the forces in these regions would not be evaluated as unstable forces. The milling of various volumes for a given processing direction across the three regions was evaluated.

### 3.2. Evaluation of Cutting Strategies

**Figure 10:**
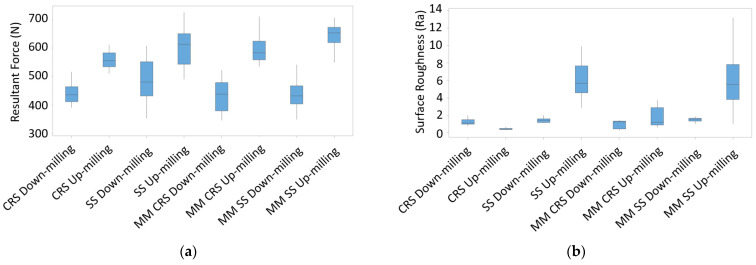
(**a**) Resultant force and (**b**) surface roughness results for up- and down-milling.

To determine whether up- or down-milling produces a better surface, the two cutting strategies were first compared regarding the forces they produced (see Figure 10a). Figure 10a shows that up-milling produced much higher forces than down-milling for all material conditions. This was expected due to the characteristics of this milling strategy. However, high forces do not necessarily produce high surface roughness. Therefore, the surface roughness for up- and down-milling was investigated for each material. The results are shown in Figure 10b. Except for the CRS up-milling case, up-milling produced significantly worse surface quality than down-milling in all cases.

**Figure 11:**
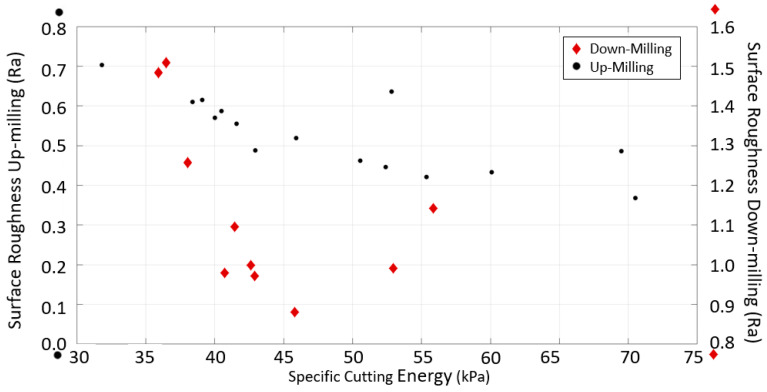
Surface roughness vs. specific cutting energy correlation for sample 4 made of CRS material.

It is unclear whether up-milling or down-milling produces better surface quality because of the variation in volume, especially for the up-milling of CRS. The specific cutting energy was thus studied to determine the best milling strategy. Contrary to the literature, better surface qualities were obtained with up-milling at similar specific cutting energy levels, as shown in Figure 11. Note that similar results have been reported in the literature [12,24]. This may be due to the thickness of undeformed chips, which is not included in the specific cutting energy calculation. The undeformed chip thickness for up-milling is smaller than for down-milling [24]. In addition, the deflection for down-milling is higher than that for up-milling. According to the time-domain displacement signals, more chatter vibration occurred during down-milling. Therefore, up-milling produced parts with better surface quality.

**Figure 12:**
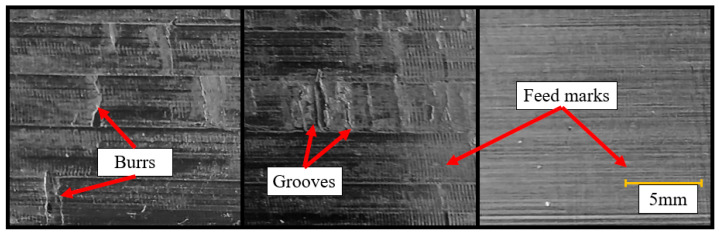
Macro dents on machined surfaces obtained with up-milling.

However, up-milling caused severe dents in SS, as shown in Figure 12. Therefore, although up-milling outperformed down-milling in terms of specific cutting energy, down-milling produced better surface quality. Further evaluations were thus conducted with down-milling.

### 3.3. Evaluation of Axial Depth of Cut

**Figure 13:**
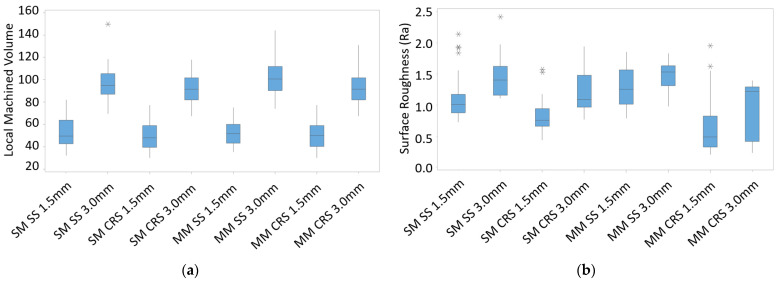
(**a**) Calculated local machined volume and (**b**) surface roughness results for various axial depth cut values.

This study used two axial depths of cut values, 1.5 and 3 mm, for machining the parts. As shown in Figure 13a, although the 3 mm axial depth of cut removed more local machined volume in general, the local machined volumes for the two axial depth of cut values were similar for most cases. The surface roughness due to the fact of machining was also compared for the two axial depths of the cut. As shown in Figure 13b, there was no apparent difference in the surface roughness between the two axial depth of cut values. For box plot preparation, the upper limit is obtained when the third quartile value and 1.5 times the interquartile range value are summed. According to the limit, extreme outliers are marked with an asterisk (*) on the boxplot. The local actual machined volume was thus used instead of the axial depth of cut in this study.

### 3.4. Evaluation of Surface Irregularity

**Figure 14:**
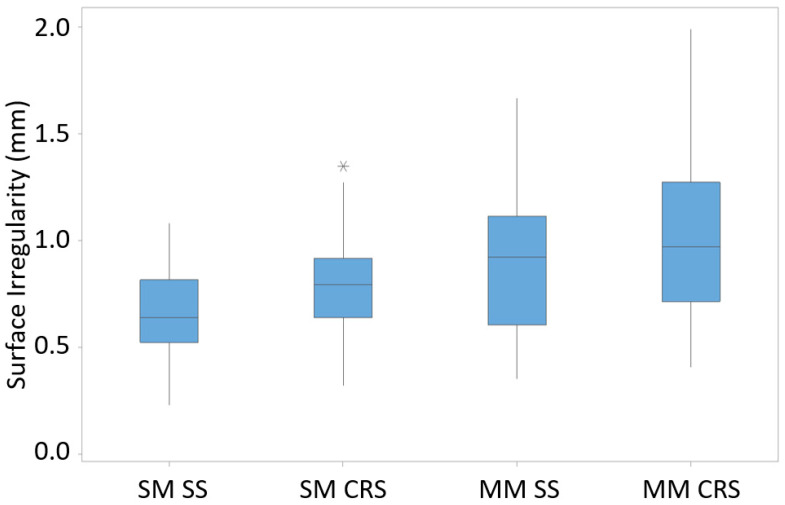
Surface irregularity for various components (SM: single-material; MM: multi-material).

The surface irregularity of CRS was higher than that of SS, as shown in Figure 14. Although the production was conducted with similar heat inputs, the Cr content in CRS was much higher than in SS, which might explain this result. It is known that Cr increases the flowability [25]. The irregularity of the CRS parts was thus high. In addition, the irregularity of the multi-material (SS + CRS) component was found to be much higher due to the higher heat input, as shown in Table 2.

### 3.5. Evaluation of the Resultant Force and Local Machined Volume for Single Materials

**Figure 15:**
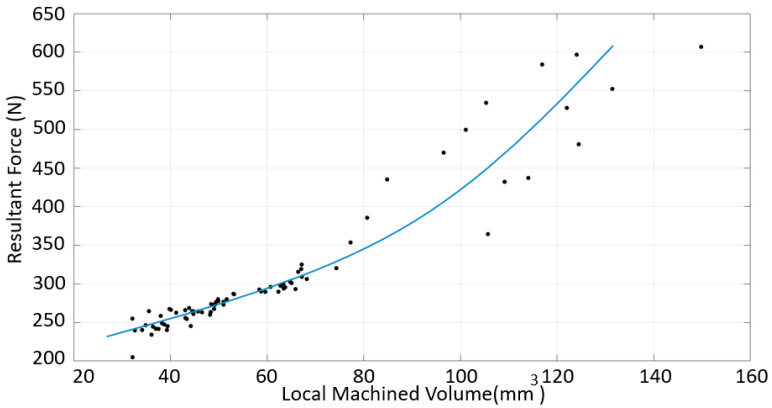
Resultant force vs. volume for the SS material, down-milling cases.

No correlation was established between the force data collected in each region and surface roughness. Furthermore, no correlation was established between the irregularity in a region and surface roughness. The resultant force and machined volume were thus evaluated in an intermediate step. As shown in Figure 15, the resultant force increased with the increasing volume for the down-milling of SS. However, it can be seen that although there was an almost linear increase up to approximately 70 mm^3^, the correlation was not maintained at high volumes.

**Figure 16:**
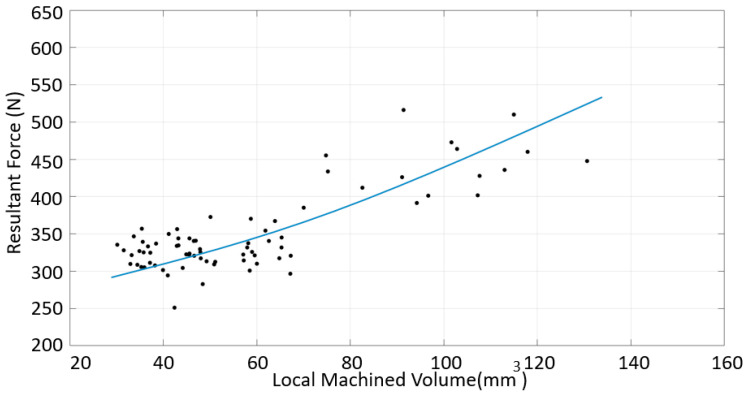
Resultant force vs. volume for the CRS material, down-milling cases.

A similar analysis was performed for the down-milling of CRS. As shown in Figure 16, the resultant force increased with the increasing volume, as was the case for SS. However, the correlation was not as clear as that for SS. This could be due to the irregularity of the surface.

### 3.6. Evaluation of the Specific Cutting Energy and Surface Roughness for Single Materials

Although a correlation was found between the resultant force and volume, no clear correlation was found between the resultant force and surface roughness. Therefore, an attempt was made here for WAAMed parts with high surface irregularity to find a correlation between the specific cutting energy and surface roughness. The specific cutting energy *U*, expressed in Equation (2), was calculated using the resultant force and machined volume values (Equation (3)). A few studies have related surface defects to surface roughness due to the fact of machining using a specific cutting energy [13,23,24,26]. In Equation (3), *F_x_* is the axial force, *F_y_* is the radial force, *V_c_* is the cutting speed, and *MRR* is the material removal rate. As described in the literature [23], the feed rate was not included in the calculation of the specific cutting energy, because its value is low enough to be ignored; instead, the cutting rate was used.

**Figure 17:**
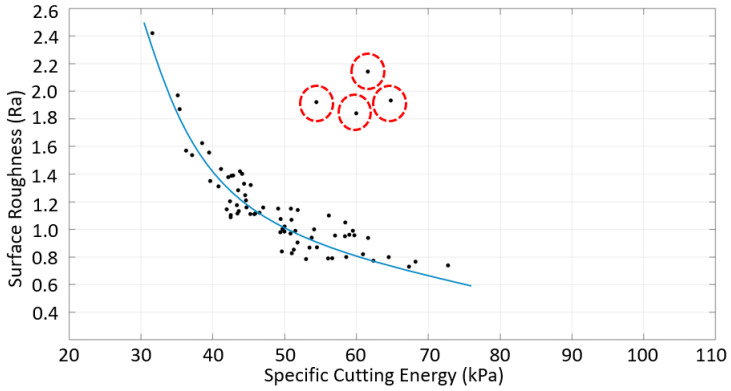
Surface roughness vs. specific cutting energy for the down-milling of SS (red circles indicate outliers).

As shown in Figure 17, the surface roughness decreased with the increasing specific cutting energy for the down-milling of SS. Previous studies have reported this trend on specific cutting energy [23,24]. However, there were some outliers to the trend, as indicated by the red circles in the figure. This may be due to the fact of chatter vibration and high surface irregularity. These outliers are explained in more detail in the next section.

(2) $$U\left(kPa\right)=\frac{P_{c}\;\left(W\right)}{MRR\;\left(mm^{3}/s\right)}$$

(3) $$P_{c}\left(W\right)=\left(\sqrt[{\;}]{F_{x}^{2}+F_{y}^{2}}\left(N\right)\right)\times V_{c}\left(m/s\right),$$

**Figure 18:**
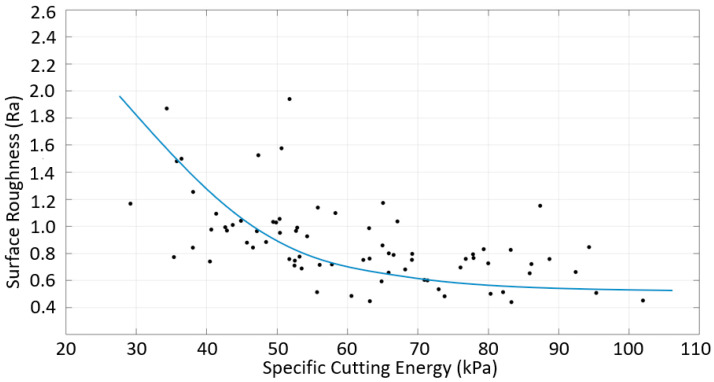
Surface roughness vs. specific cutting energy for the down-milling of CRS.

A similar study was conducted for the down-milling of CRS. Figure 18 shows that the surface roughness decreased with the increasing specific cutting energy, as was the case for SS. A comparison of Figure 15 and Figure 16 indicates that the higher surface quality for CRS can be explained by the specific cutting energy, although higher forces were produced during the machining of CRS. A comparison of Figure 17 and Figure 18 indicates that the specific cutting energy for CRS was much higher than that for SS, so the surface roughness of CRS was better. These figures show that the correlation between the specific cutting energy and surface roughness for the down-milling of SS was slightly different from that for CRS. In other words, different surface roughness values were found for a given specific cutting energy. This situation is related to surface irregularity; cases with a high roughness that results in a high specific cutting energy have high irregularity.

### 3.7. Evaluation of Multi-Material Machining

**Figure 19:**
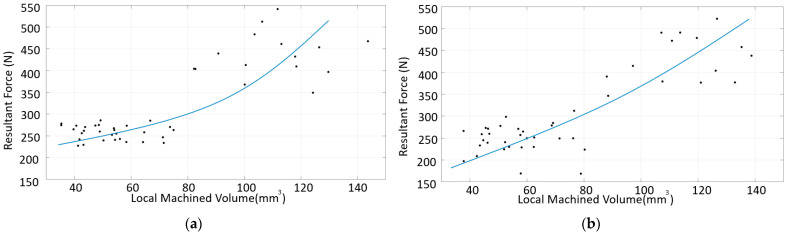
Resultant force vs. volume for the down-milling of the multi-material component with (**a**) SS machined and (**b**) CRS machined.

Some general trends for the volume and force, specific cutting energy, and surface roughness for SS and CRS were identified above. Similar trends were found at low volume and low irregularity for multi-material (SS + CRS) components, regardless of which side, SS or CRS, was machined. Figure 19a,b show similar results for the multi-material components as for the single-material components. However, unlike for the single-material components, different forces were found at similar volume values for the multi-material components.

**Figure 20:**
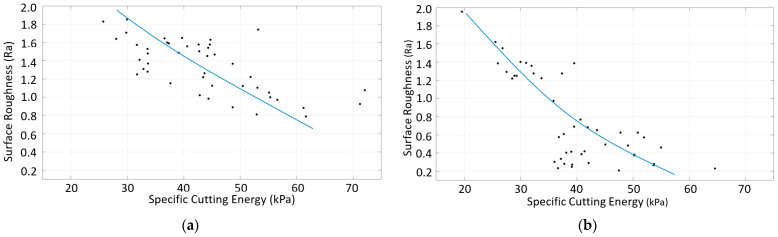
Surface roughness vs. specific cutting energy for the down-milling of multi-material components with (**a**) SS machined and (**b**) CRS machined.

Figure 20 shows the relationship between surface roughness and specific cutting energy for multi-material components with either SS or CRS machined. Although similar trends were obtained, very different surface roughness values were obtained for the specific cutting energy for the two-component types. This may be due to the differences in irregularity. In addition, for the single-material components, the surface roughness of CRS was better than that of SS due to the higher specific cutting energy for CRS. However, different surface roughness values were obtained at similar specific cutting energies for the multi-material components.

**Figure 21:**
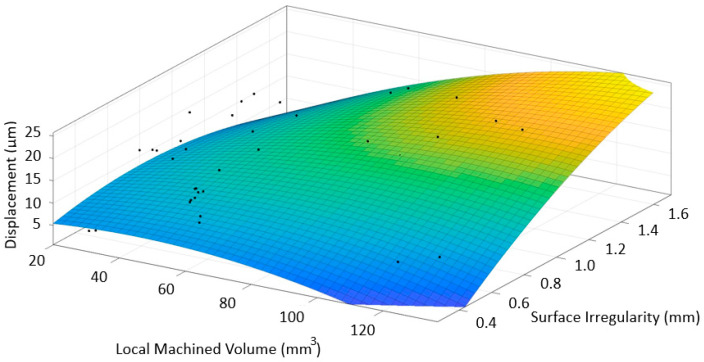
Correlation between displacement, machined volume, and surface irregularities.

These results may be related to surface irregularity. The waviness of the multi-material deposits was higher than that of SS, and the CRS deposited as single materials. This is due to the extra heat input for the multi-material deposits [8]. Although the displacement data did not show a general trend, it was observed that the surface quality deteriorated with the increasing time-domain displacement signal values, as expected. As shown in Figure 21, low irregularity caused low displacement even if a high volume was processed. However, with increasing irregularity, the displacement increased independent of the volume. In the worst case, both irregularity and volume were high. In this case, high displacement was obtained. High displacement usually indicates a marginally stable case and/or areas with chatter, with chatter marks or dents appearing on the surface. The surface quality thus deteriorates.

### 3.8. Evaluation of Instability Situations

The movement of the workpiece due to the fact of vibration during machining was recorded using a proximity probe. It was found that the surface roughness deteriorated at points with high displacement, as expected. In addition, the presence of chatter could be determined from the proximity probe sensor data. Although this method is not commonly used in the literature, it has been validated [8].

**Figure 22:**
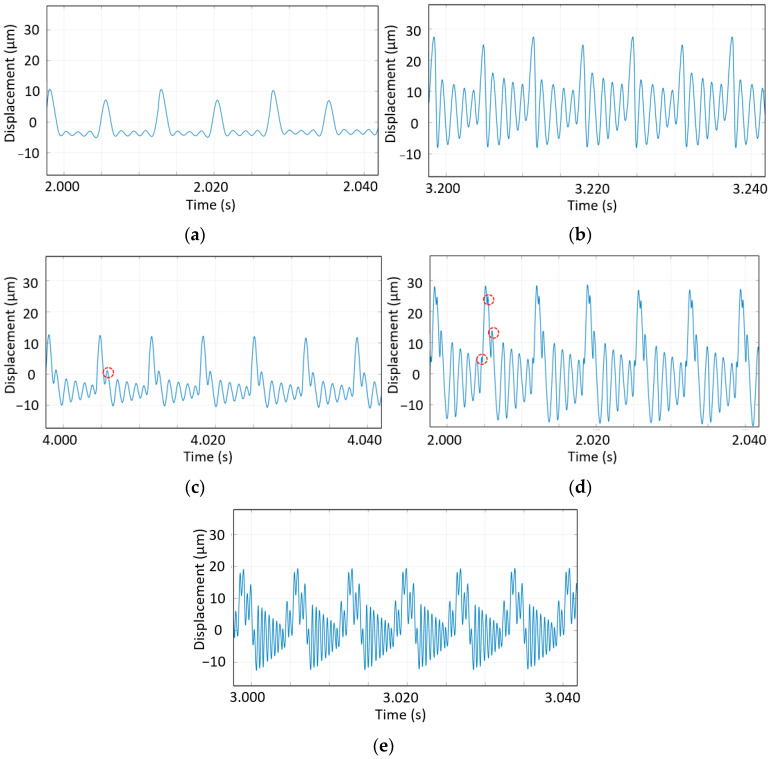
Chatter vibration for (**a**) sample 3, (**b**) sample 5, (**c**) sample 6, and (**d**,**e**) sample 7 (red circles indicate peaks caused by chatter).

According to the literature [8], the displacement data should peak when the insert penetrates the part; it should then oscillate and almost disappear until the other insert penetrates the part. This cycle is then repeated, as shown in Figure 22a,b. Note that there was no chatter vibration. The magnitudes of the alternating peaks were different, which may be caused by the runout. On the other hand, for the data with outliers shown in Figure 17, regions with chatter vibration formed, as shown in Figure 22c,d.

In these figures, after the displacement peaked, it peaked again at the outlier points before reaching the second peak. This suggests that chatter vibration occurred at these points. However, further study of the chatter is required to verify this. Although a correlation between the specific cutting energy and surface roughness was obtained, it did not hold for cases with chatter. The surface irregularity and volume for parts with chatter vibration were also examined, but no correlation was found.

### 3.9. Evaluation of Hardness Measurements

**Figure 23:**
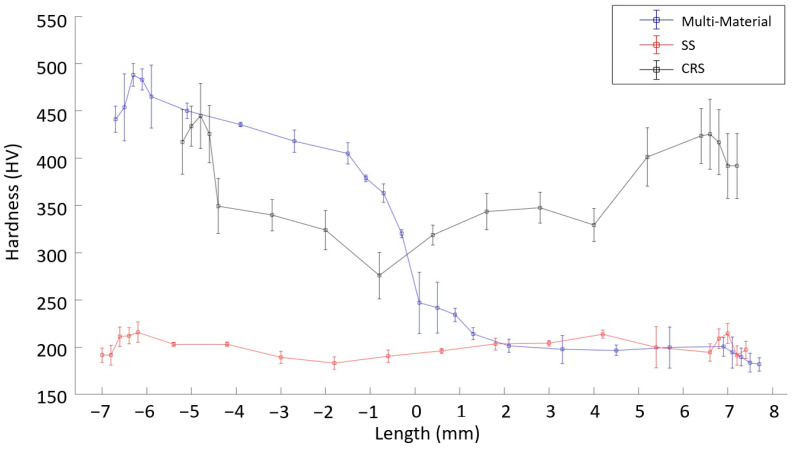
Hardness variation throughout various components (length of 0 indicates midpoint).

The start and end parts of the WAAM deposition were separated from the part before machining. Hardness measurements were carried out for the as-built form (i.e., the previously separated parts). These measurements focused on the edges, as an average of 1 mm of material was removed during processing. The hardness measurement results for single- and multi-material components are shown in Figure 23. A length of zero indicates the midpoint of the parts. Since the parts had different thicknesses, the distances of the end parts were different.

As shown in Figure 23, the hardness was in the order of multi-material component > SS > CRS at the edges. The deposits were constructed by making two deposits side by side in one layer, as shown in Figure 1. Since two deposits are made in a single layer, they intersect. According to the distance data shown in Figure 23, the interval from approximately −2 to 2 mm represents the intersection of the two deposits. For both SS and CRS, there was a decrease in the hardness in the intersection region due to the increased cooling time. In addition, the decrease in the hardness in the intersection region for CRS was much larger than that for SS. This may be related to the thermal conductivity difference between these two materials. Because the thermal conductivity of CRS is lower than that of SS, the cooling process takes longer for CRS, and accordingly, the decrease in the hardness at the intersection is much larger than that for SS.

The transition from CRS to SS based on hardness is quite clear in the intersection region for the multi-material component. Of note, the CRS side of the multi-material component had a higher hardness than that of the single-material CRS component. This can be explained as follows. First, the heat input for the single-material component is higher than that for the multi-material component and, thus, the former is softer during the deposition. The SS samples verify this; the heat input for the multi-material deposit on the SS side was higher than that for the single-material SS component. As a result, the multi-material component with SS machined had a lower hardness than that of a single SS. In addition, as explained in the comparison of single-material components, the lower thermal conductivity of CRS may explain the lower hardness of the multi-material component. For the multi-material deposits, CRS was deposited before SS. The CRS side was exposed to extra heat input during the deposition of the SS side, and the process of removing the total heat from the CRS side was extended. The cooling time for the CRS side of the multi-material component may thus be longer than that for the single-material CRS component. This may have caused the part to be softer and have a longer cooling time.

It is expected that higher forces can be obtained in the machining of hard materials. Therefore, high forces in the machining of hard materials are expected for a given machined volume. This is generally confirmed by the fact that higher forces were obtained during the machining of CRS for lower machined volumes. However, there was no difference between the forces of SS and CRS for higher machined volumes, even though they have an almost two-fold difference in hardness. As explained in previous sections, this general trend did not hold for a given volume due to the fact of high surface irregularity. Similar forces were obtained for both SS and CRS for high machined volumes. In short, the effect of the hardness on machining was not observed due to the high surface irregularity. Hardness should thus not be used as a criterion for the machinability of as-built surfaces of WAAMed parts.

## 4. Conclusions

The present study produced single-material (SS or CRS) and multi-material (SS + CRS) parts with thin-walled geometries via WAAM. The produced samples were 3D scanned and milled using the calculated machining allowances. The correlations between surface roughness and the resultant force, displacement, calculated actual machined volume, and specific cutting energy were examined. Comparative analyses were performed to determine the effect of the machining strategy on the surface roughness. In addition, correlations were established between the specific cutting energy and surface roughness. The experimental results can be summarized as follows:

1. Due to the high surface irregularity of WAAMed parts, machining with a constant volume and a constant material removal rate is not possible even if constant axial and radial depths of cut are applied to machine parts under stable conditions.
2. Cutting forces increase with an increasing machined volume, but there is no correlation between cutting forces and surface roughness.
3. Specific cutting energy is an important criterion for producing low surface roughness. Similar surface roughness can be achieved by applying both high and low cutting forces.
4. The specific cutting energy was calculated from the resultant force and actual machined volume. An inverse relationship was obtained between the specific cutting energy and surface roughness.
5. There was no difference in the machinability for SS, CRS, and multi-material components for machining with low volume and surface irregularity. In other words, depositing single- or multi-material leads to no difference in the machinability for low flatness/waviness and low volume.
6. High irregularity and high volume negatively affect the machinability regardless of the material type.
7. Machining allowances calculated from 3D scanning data cannot be used as radial depth of cut values to achieve low surface roughness.
8. The axial depth of the cut loses its significance due to the high surface irregularity. It was observed that more chips were removed with a depth of cut value of 1.5 mm than a value of 3 mm due to the fact of high surface irregularity.
9. The radial depth of the cut should be chosen according to the predicted machined volume, force, and specific cutting energy.
10. The heat input for the deposition has a large effect on the hardness of the surfaces to be machined.
11. For machining the as-built surfaces of WAAMed parts, a high hardness does not necessarily cause high forces due to the fact of high surface irregularity. Hardness should thus not be used as a criterion for evaluating the machinability of as-built surfaces.

## Figures and Tables

**Table 1:** Chemical composition of the filler materials (%).

Material	Designation	C	Si	Mn	Cr	Mo	Ni
Stainless steel	EN 12072: G2312L AWS 5.9: ER309 L	0.02	0.5	1.7	24.0	-	13.2
Creep resistant steel	EN 12070: G CrMo2 Si AWS 5.28: ER90S-G	0.06	0.7	1.1	2.6	1.0	-

**Table 2:** WAAM deposition parameters and calculated heat inputs.

Material	Deposition Strategy	Wire Feed Speed (m/min)	Travel Speed (cm/min)	Heat Input (kJ/mm^3^)
Stainless steel	Single-material	4.9	40.2	0.018
Creep-resistant steel	Single-material	2.3	22.1	0.022
Stainless steel	Multi-material	3.5	25.6	0.028
Creep-resistant steel	Multi-material	3.4	20.3	0.016

**Table 3:** Milling parameters used for CRS and SS.

Material	Cutting Speed (m/min)	Feed Rate (mm/Tooth)
CRS	265–280	0.1
SS		

**Table 4:** Machining allowances calculated based on the 3D scanning results.

Sample No.	Machining Allowance (mm)
1	1.59
2	1.95
3	2.55
4	1.79
5	1.76
6	2.05
7	2.58

**Table 5:** Experimental settings used for milling.

Sample No.	Material	Cutting Strategy	Axial Depth of Cut (mm)
1	Multi-material	Up-milling	3.0
2	Multi-material	Down-milling	3.0
3	Multi-material	Down-milling	1.5
4	CRS	Up- and down-milling	3.0
5	CRS	Down-milling	1.5
6	SS	Up- and down-milling	3.0
7	SS	Down-milling	1.5

## Data Availability

The raw/processed data generated during and/or analyzed during the current study are available from the corresponding author upon reasonable request.
